# Enhancing allergy diagnosis: mass spectrometry as a complementary technique to the basophil activation test

**DOI:** 10.3389/falgy.2025.1568670

**Published:** 2025-05-30

**Authors:** Nicole Wheeler, Miloslav Sanda, Lanya Rasool, Noha Elemary, Arda Alpan, Hamed Safi, Denise Loizou, Matthew Plassmeyer, Mikell Paige, Soren Ulrik Sonder, Oral Alpan, Michael Girgis

**Affiliations:** ^1^ Department of Chemistry & Biochemistry, College of Science, George Mason University, Fairfax, VA, United States; ^2^ Max-Planck-Institute for Heart and Lung Research, Bad Nauheim, Germany; ^3^ Department of Clinical Research, Amerimmune LLC, McLean, VA, United States; ^4^ Department of Flowcytometry, Amerimmune LLC, McLean, VA, United States; ^5^ Center for Molecular Engineering, George Mason University, Manassas, VA, United States; ^6^ Department of Bioengineering, College of Engineering & Computing, George Mason University, Fairfax, VA, United States

**Keywords:** BAT, mass spectrometry, food allergy, diagnostics, specificity, sensitivity

## Abstract

Accurate diagnostic tools for allergic conditions are essential for effective treatment. Traditional methods, such as skin prick tests (SPT) and specific IgE measurements are widely used, but they have limitations in sensitivity and specificity for certain allergens. While the Basophil Activation Test (BAT) offers improved specificity, particularly for allergens such as peanuts and sesame, its practicality and accessibility remain challenges. Mass spectrometry (MS) has recently gained recognition as a promising complementary tool in allergy diagnostics, offering high analytical precision and the capability to detect a wide range of allergen-specific biomarkers. This review explores the integration of MS into allergy diagnostics, emphasizing its potential to enhance BAT applications, particularly for non-responders. We discuss the underlying mechanisms, recent research highlighting its efficacy, and the technical challenges that must be addressed for clinical adoption. Additionally, we examine the standardization requirements and ethical considerations necessary for MS to become a routine diagnostic tool. Finally, we consider the future of allergy diagnostics, highlighting how MS technology could contribute to more precise, personalized, and patient-centered care in allergy management.

## 1 Introduction

Food allergic reactions begin when the immune system mistakenly identifies a harmless food protein as a threat. This leads to the activation of B-cells (plasma cells), which produce allergen-specific Immunoglobulin E (IgE) antibodies (1–3). These antibodies then bind to high-affinity Fc*ε*RI receptors on the surface of mast cells and basophils, a process known as sensitization. Upon subsequent exposure, the allergen cross-links the IgE antibodies on mast cells and basophils, triggering cellular activation and degranulation. During an IgE-mediated allergic reaction, the immune activation leads to the release of both pre-formed mediators stored in granules and newly synthesized molecules that contribute to the inflammatory response. Pre-formed mediators, such as histamine and tryptase, are rapidly released upon mast cell and basophil degranulation. Histamine plays a central role in the immediate hypersensitivity response by inducing vasodilation, increased vascular permeability, smooth muscle contraction, and pruritus. Tryptase, primarily secreted by mast cells, serves as a biomarker of mast cell activation and contributes to tissue remodeling and inflammation. In contrast, newly synthesized mediators, including leukotrienes, cytokines, and prostaglandins, are produced *de novo* following cellular activation (4–8). Leukotrienes, such as LTC₄, LTD₄, and LTE₄, contribute to prolonged bronchoconstriction, mucus secretion, and increased vascular permeability. Key cytokines, including IL-4, IL-5, IL-13, and TNF-α, regulate immune responses by promoting eosinophil recruitment, enhancing IgE production, and sustaining chronic inflammation. Additionally, prostaglandins, particularly Prostaglandin D₂ (PGD₂), play a role in bronchoconstriction, vasodilation, and immune cell recruitment (9, 10). Collectively, these mediators drive both the early-phase and late-phase allergic responses (11–15). These biochemical changes manifest clinically as allergic symptoms, ranging from mild reactions such as skin rashes, itching, and nasal congestion to severe respiratory distress, gastrointestinal issues, and life-threatening anaphylaxis.

**Figure 1:**
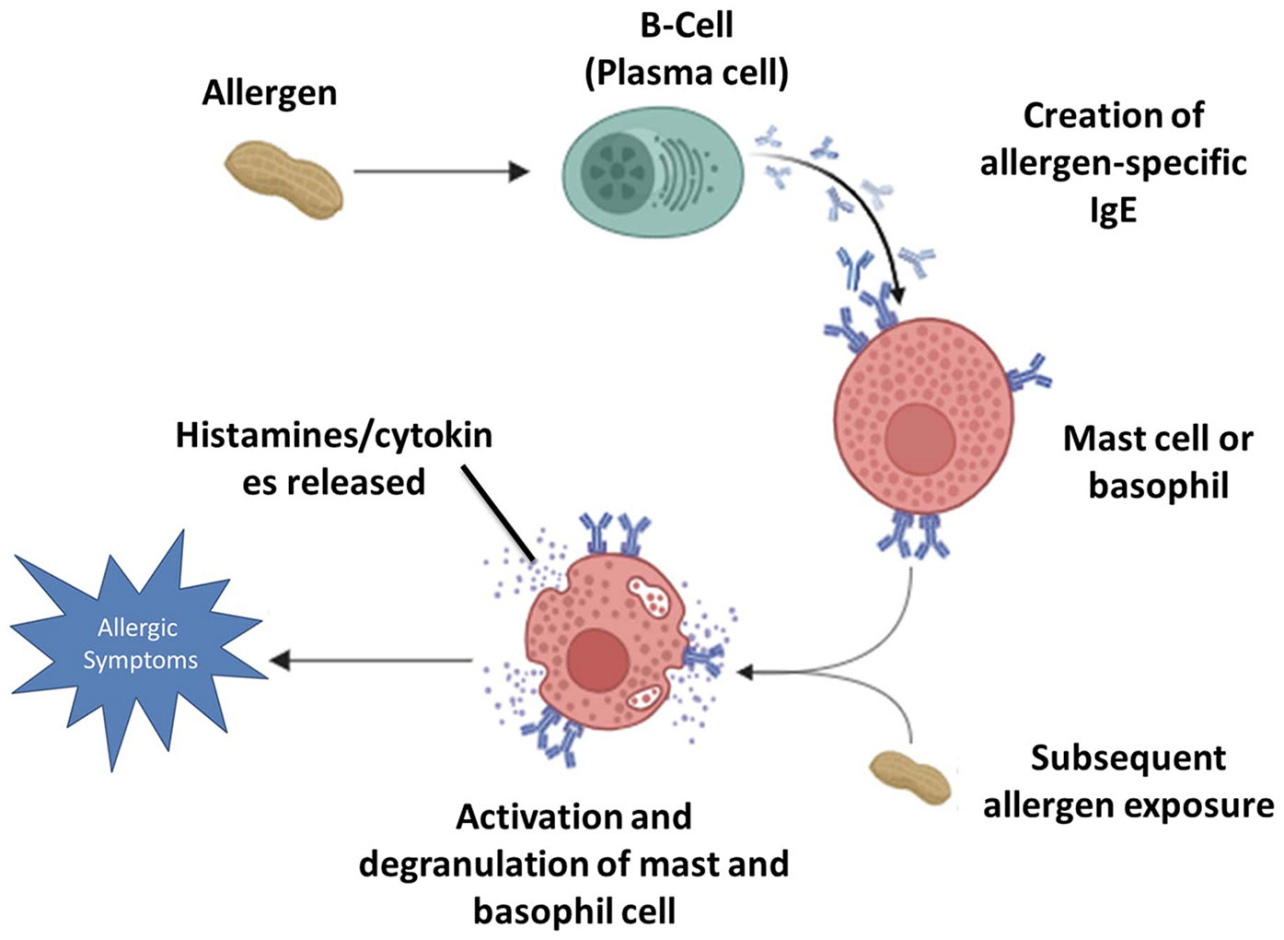
The mechanism of IgE-mediated allergic reaction.

The IgE-mediated responses tend to be immediate, causing a rapid onset of symptoms, while non-IgE-mediated reactions typically manifest later (2, 16, 17). Figure 1 depicts the mechanism of IgE-mediated allergic reactions described above.

The Müller classification categorizes allergic reactions into four grades based on severity, providing a structured framework for clinical assessment. A Grade I reaction includes mild symptoms such as skin flushing, urticaria, and mild angioedema without respiratory or cardiovascular involvement. Grade II reactions are moderate and involve more pronounced symptoms, such as difficulty breathing, wheezing, nausea, and mild hypotension, but without life-threatening manifestations. Grade III reactions are severe and, characterized by life-threatening airway obstruction, bronchospasm, or severe hypotension. Grade IV represents the most critical cases, with circulatory and respiratory failure (16, 18, 19).

The IgE-mediated reactions, in particular, are associated with the most severe cases of food allergies, emphasizing the critical need for accurate diagnosis and effective management to prevent accidental exposure to allergens (20–22). Therefore, IgE-mediated food allergy diagnosis will be our focus in this study.

The gold standard of food allergy diagnosis is the oral food challenge (OFC). The intentional exposure to multiple allergens in OFCs introduces a considerable risk to patients, as it inherently involves provoking allergic reactions (23).

Other diagnostic techniques like the Skin Prick Test (SPT), Specific IgE (sIgE) testing, and the Basophil Activation Test (BAT), are known to be ineffective or inaccurate in some situations. They often require supplementation with patient-reported symptoms or clinical history, which increases the risk of mischaracterizing food sensitivities as true allergies, leading to inappropriate and false-positive diagnoses (24–28).

The SPT test involves the application of small amounts of potential allergens to the skin, typically on the forearm. Although it is generally safe, it can have issues, such as the possibility of false positives, especially with certain foods. While elevated IgE levels alone do not confirm sensitization, higher IgE concentrations are associated with an increased likelihood of clinical allergy and greater severity of allergic reactions. Therefore, it must be used in conjunction with other diagnostic methods to provide a more comprehensive assessment (29–32).

The sIgE testing is a valuable diagnostic tool used to measure allergen-specific immunoglobulin E antibodies in the bloodstream, enabling the identification of sensitization to particular allergens. This method is especially effective for diagnosing IgE-mediated (Type I) hypersensitivity reactions, including those related to food, environmental allergens, and insect venom. Unlike SPT, sIgE assays carry no risk of provoking an allergic response, making them suitable for patients with dermatologic conditions, those on antihistamines, or individuals unable to undergo skin testing. Commonly employed platforms, such as ImmunoCAP and Immulite, provide quantitative assessments of allergen-specific IgE levels, supporting clinicians in the evaluation of allergy severity, monitoring of therapeutic interventions, and formulation of targeted management strategies (33, 34).

The BAT demonstrates high sensitivity and negative predictive value (NPV) across various food allergens, making it a reliable diagnostic tool. For peanut allergy**,** BAT shows 75% sensitivity and 98% specificity, effectively distinguishing allergic individuals. In cow's milk allergy, it has 89% sensitivity**,** 83% specificity, and a 96% NPV**,** strongly predicting tolerance when negative. For egg allergy, BAT's sensitivity ranges from 63%–77%, with 96%–100% specificity, particularly when assessing CD63 expression. These values highlight BAT's role in accurately ruling out allergies and reducing unnecessary dietary restrictions (35–37).

**Figure 2:**
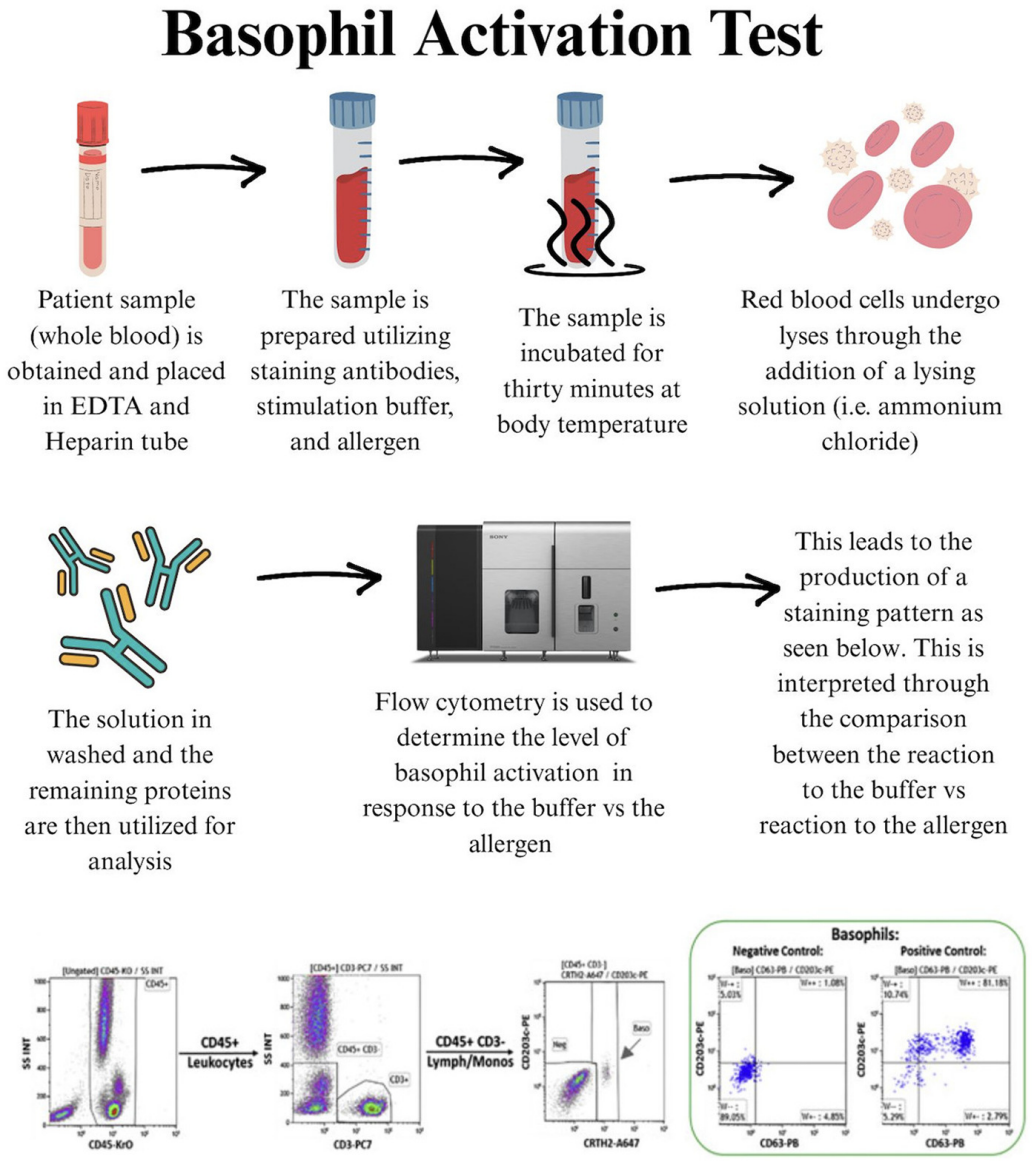
The workflow for BAT test.

Also, the BAT is a functional diagnostic tool that evaluates the activation of basophils in response to allergens. This test utilizes flow cytometry to measure the translocation of activation markers to the surface of the basophils when exposed to specific allergens. The BAT offers a more specific alternative to sIgE and SPT by directly measuring cellular reactivity to allergens. The BAT has the advantage of reducing false positives and better predicts clinical reactivity, potentially enhancing diagnostic accuracy in food allergy assessments (38–41, 42). The test works by exposing basophils in a blood sample to allergens and detecting surface markers such as CD63 and CD203c via flow cytometry, signaling an allergic response (43, 44). Figure 2 illustrates the typical workflow for the BAT test. Despite the increased specificity of BAT, 15%–20% of people tested have basophils that fail to respond to IgE-mediated stimulation, likely due to a mutation in the promoter of the SYK gene (45).

The SPT and specific sIgE tests serve as first-line diagnostic tools for food allergies; however, both have limitations in specificity, which can lead to false-positive results. The BAT, a second-line diagnostic tool, offers improved specificity by directly assessing cellular reactivity, yet it may still be affected by basophil non-responsiveness in certain individuals. The introduction of component-resolved diagnostics (CRD) has significantly enhanced specificity by identifying allergen-specific IgE at the molecular level, thereby reducing misclassification and improving diagnostic accuracy when used in conjunction with BAT tests.

The Basophil Activation Test (BAT) does not always exhibit high sensitivity, with values varying across allergens. For Example, peanut allergy sensitivity is around 75%, cow's milk allergy reaches 89%**,** while egg allergy can be as low as 63% (46). However, BAT's specificity is particularly high often exceeding 95%**,** making it highly valuable when skin prick tests (SPT) and specific IgE levels yield inconclusive results in the “gray zone.” For peanut allergy, BAT has 98% specificity, for egg allergy 96%–100%, and for cow's milk allergy around 83%, ensuring fewer false positive results and reducing the need for oral food challenges (OFC), which, while effective, can be burdensome and carry inherent risks (39, 47, 48).

Mass Spectrometry (MS) technology is increasingly employed in medical diagnostics due to its high sensitivity, specificity, and ability to analyze complex biological samples with great precision. For instance, tandem mass spectrometry (MS/MS) plays a vital role in newborn screening by analyzing dried blood spots to detect metabolic disorders such as phenylketonuria (PKU) and other inborn errors of metabolism with high accuracy and sensitivity (49–51). Furthermore, MS played a vital role during the COVID-19 pandemic by aiding in the structural analysis of SARS-CoV-2 proteins, which contributed to the development of diagnostics and therapeutics, showcasing its versatility in addressing emerging global health challenges (52–54).

One promising application of MS is in food allergy diagnostics, where its enhanced sensitivity, specificity, and high-throughput analytical capabilities could significantly improve the detection and characterization of allergenic reactions. It is estimated that more than 10% of the US population suffers from at least one food allergy, with rates continuing to rise globally (7). Given the challenges of current diagnostic tools, there is a dire need for more accurate and reliable methods for allergy diagnosis which represents an opportunity to leverage MS's unparalleled specificity (55). Through direct identification and quantification of allergens and specific antibodies such as IgE based on their unique mass-to-charge ratios, MS has the potential to offer insights into individual sensitivities and immune responses.

Although clinical applications of MS are still developing, it holds promise for advancing the management of food allergies. This review examines the potential application of MS in food allergy diagnostics, focusing on its role in quantifying immunoglobulins such as IgE and its advantages over current assays. Additionally, we explore how MS could enhance diagnostic precision and enable personalized allergy management, addressing existing gaps in sensitivity, specificity, and clinical applicability in food allergy testing.

## 2 Time dependent response of basophils

It was demonstrated that the BAT, performed by measuring basophil surface markers and intracellular phosphorylation of signaling molecules over a 20-min period at intervals ranging from seconds to several minutes (e.g., 1, 3, 5, 10, and 20 min, or other combinations), correlates with the severity of allergic reactions. Allergens are applied at varying concentrations ranging from 0.1 to 100,000 ng/ml.

First, whole blood is collected from subjects and incubated with the allergen for varying durations and concentrations. Blood cells are subsequently analyzed for the expression of cell surface markers, such as CD203c and CD63, and intracellular phosphorylation markers, including phospho-Lyn, phospho-Fyn, and phospho-IgE receptors. Remarkably, the phosphorylated targets, including the IgE receptor, Fyn, and Lyn, are proximal to Syk kinase and remain unaffected by the absence of Syk. This characteristic enables the assessment of patients with non-reactive basophils based on conventional surface markers (e.g., CD63 or CD203c), who might otherwise not respond to traditional BAT. This method is proposed for use in diagnosing or monitoring allergies and evaluating the effectiveness of therapeutic interventions based on the responsiveness of an individual being treated (37).

**Figure 3:**
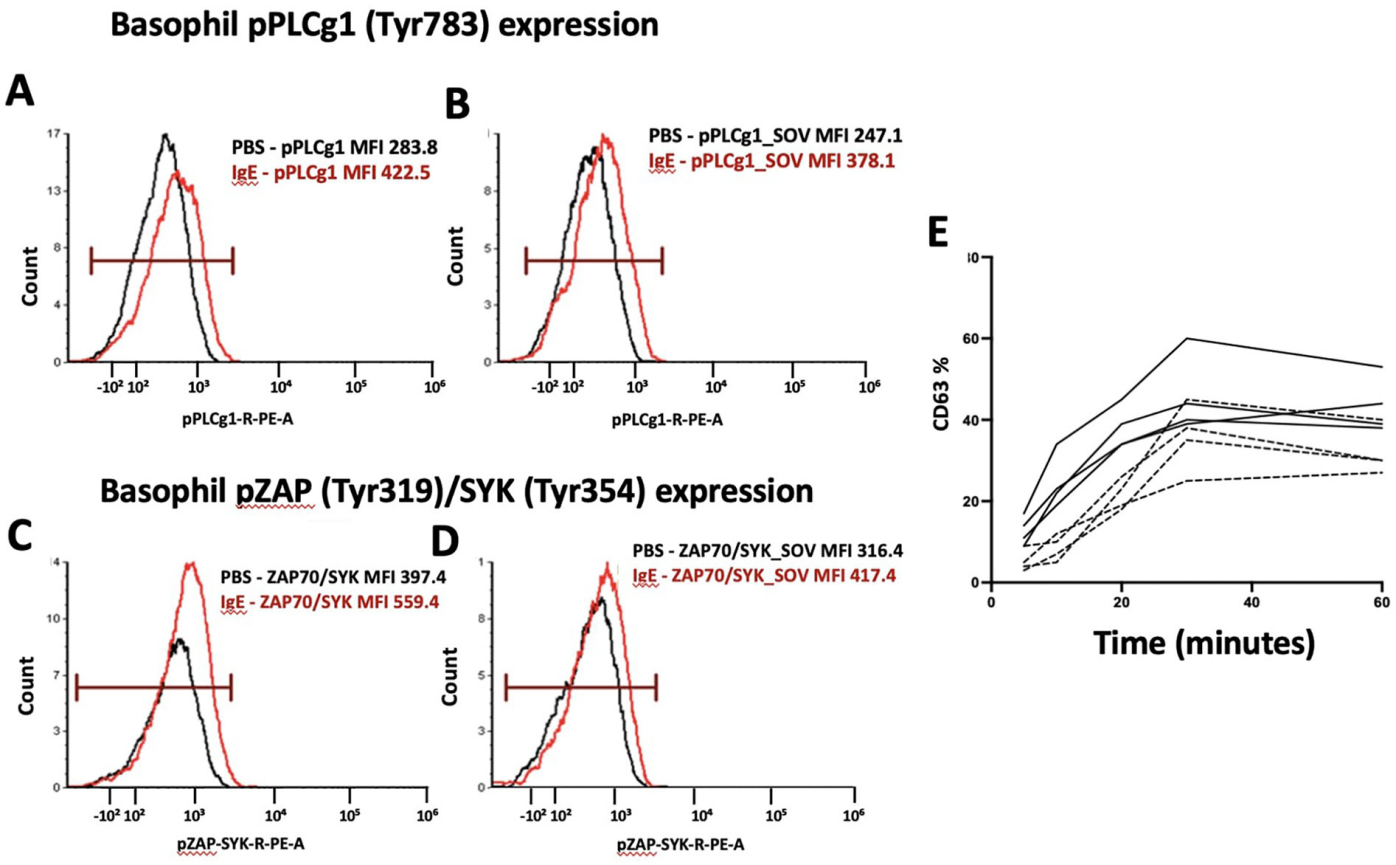
Phosphorylation of signaling proteins in basophils after IgE stimulation. **(A,B)** Expression of phosphorylated PLCγ1 (pPLCγ1, Tyr783) in basophils treated with PBS (black) vs. IgE (red). **(C,D)** Expression of phosphorylated ZAP70/SYK (pZAP, Tyr319/Tyr354) in basophils treated with PBS (black) vs. IgE (red). **(E)** Basophil CD63 upregulation in patients with mild peanut allergy with perioral hives (dashed line) vs. full anaphylactic reaction (solid line) requiring epinephrine.

A key advantage of this approach is its ability to address the issue of non-releaser or low-responder basophils, observed in 10%–20% of the population (35, 56). Additionally, measurement of the rate of upregulation of these markers over short time intervals introduces a novel aspect, enhancing the sensitivity and specificity of BAT for determining clinical reactivity. Furthermore, an inhibitor of basophil activation (btk inhibitor Ibrutinib) can be used. Btk inhibitors inhibit the activation of the IgE stimulation pathway. This assesses if basophil activation induced by the allergen is specific, as the inhibitor solely impacts the IgE pathway but not the fMLP (*N*-Formylmethionyl-leucyl-phenylalanine) induced basophil activation. FMLP is a positive control used to assess the response of basophils to a non-IgE pathway stimulation, and overall basophil reactivity. Figure 3 shows the phosphorylation of basophil phospho-proteins upon stimulation by PBS or anti-IgE (3 A-D) and CD63 upregulation over a range of incubation times (3 E). We are currently exploring the value of phospho-markers as well as the time course of the BAT assay in larger cohorts of milk, peanut, and egg allergy.

**Figure 4:**
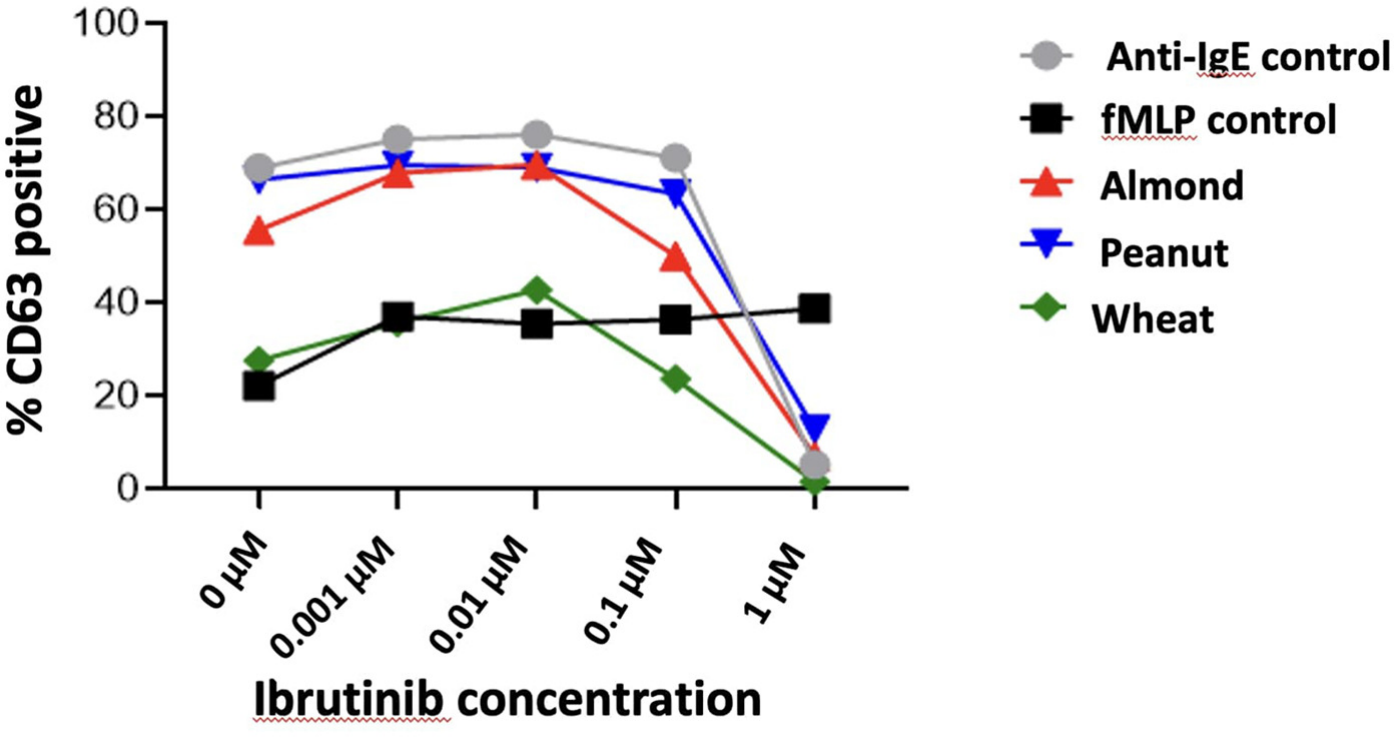
Effect of Ibrutinib on basophil activation and CD63 surface expression across different stimuli.

Basophil activation through the IgE pathway can be inhibited by the btk inhibitor Ibrutinib. This allows the assessment for the non-specific (e.g., non-IgE-dependent) stimulation of basophils, as fMLP stimulation of basophils is not affected by btk inhibitor ibrutinub and other btk inhibitors at concentrations above 0.01 µM. Figure 4. demonstrates the inhibitory effect of Ibrutinib, a BTK inhibitor, on basophil activation as measured by CD63 surface expression. The percentage of CD63-positive basophils decreases with increasing Ibrutinib concentrations (zero to one µM) across various stimuli, including anti-IgE, fMLP, and food allergens (almond, peanuts, and wheat). At higher concentrations (one µM), Ibrutinib significantly reduces allergen-induced basophil activation, indicating its potential to suppress allergic responses.

## 3 Mass spectrometry in allergy diagnosis

Mass spectrometry (MS) is emerging as a powerful tool in allergy diagnosis, offering high sensitivity, specificity, and multiplexing capabilities to detect allergen-specific biomarkers. Unlike traditional immunoassays, MS enables precise identification and quantification of allergenic proteins, peptides, and immune mediators directly from clinical samples such as serum, plasma, or nasal secretions. It can be used to detect specific IgE-bound allergenic epitopes, characterize allergen isoforms, and measure released mediators like histamine and leukotrienes during allergic reactions. Additionally, MS allows for the detailed profiling of post-translational modifications that may influence allergenicity. Its ability to provide absolute quantification and molecular-level resolution makes MS a valuable complement to standard diagnostic tools such as skin prick testing and ELISA, particularly in complex or ambiguous cases.

### 3.1 Application of MS in immunoglobulin quantification: advancing IgG and IgE detection for allergy diagnostics

The direct quantification of IgE antibodies using MS in allergy diagnostics remains a largely unexplored area, with few studies specifically addressing this application. To date, most MS-based research on immunoglobulin quantification has focused on immunoglobulin G (IgG), which is present in higher concentrations and has more established analytical methods in human samples. The limited exploration of IgE quantification via MS can be attributed to its significantly lower serum concentration and the widespread use of standardized immunoassays, such as Enzyme-Linked Immunosorbent Assay (ELISA) for IgE detection. Both IgG and IgE are immunoglobulins with different serum concentrations and functions. The IgG is more abundant and is responsible for long-term immunity, whereas IgE is involved in allergic responses and is present at much lower concentrations in serum. Despite these differences, the methodologies used for IgG quantification via MS can be employed to quantify IgE (57–59).

Studies on IgG quantification via MS have demonstrated that specific proteotypic peptides unique to the IgG molecule can be used as proxies for the whole antibody, allowing precise measurement in biological samples. One example is the MASCALE (Mass Spectrometry Enabled Conversion to Absolute Levels of ELISA Antibodies) method, which was developed to convert ELISA-derived values into absolute concentrations of IgG by calibrating the mass spectrometry signal of IgG-specific peptides (60). In this method, peptides such as VVSVLTVLHQDWLNGK, which are unique to the IgG subclass (IgG1, IgG3, and IgG4), were used to establish calibration curves that correlate the MS signal (peak area ratio) with the actual concentration of IgG antibodies in serum samples. This method has shown high accuracy and precision (60). It was suggested that MASCALE must be used alongside ELISA to improve the interpretation of immune responses, offering more precise and standardized quantification of antibodies in clinical trials (60).

Furthermore, microflow LC-MS/MS-PRM was recruited to quantify glycopeptides in serum, targeting specific *N*-glycoforms of IgG, and demonstrating the method's capability to distinguish between eight different *N*-glycoforms of IgG together with two *O*-glycoforms of hemopexin (HPX) in relation to disease biomarkers. This study introduced a targeted microflow liquid chromatography-tandem mass spectrometry in parallel reaction monitoring mode (LC–MS/MS-PRM) method for quantifying multiple glycopeptides in unfractionated serum samples, enabling precise and simultaneous quantification of these glycoforms. The application of this assay to patients with hepatitis C virus (HCV)-induced liver fibrosis demonstrated that specific IgG and HPX glycoforms could effectively detect fibrotic disease at varying stages (61). These findings highlight the potential of LC–MS/MS-PRM assays for rapid and reproducible biomarker assessments, targeting both *N*- and *O*-glycoforms of peptides, thereby advancing the clinical application of LC–MS/MS-based diagnostics (61). Additionally, a complementary study introduced a chemoenzymatic strategy for synthesizing isotopically labeled glycopeptides of IgG1, which were incorporated into an optimized LC-MS/MS-PRM workflow. The use of stable isotope-labeled N-acetylglucosamine facilitated highly accurate quantification of IgG glycoforms, with minimal variability and enhanced sensitivity through both Electron Transfer Dissociation (ETD) and Higher-energy Collisional Dissociation (HCD) workflows (62). This approach was exemplified in a rapid (13-minute) quantification of IgG1 Fc glycoforms in COVID-19 patients. Together, these methodologies underscore the versatility and efficiency of MS in high-throughput immunoglobulin quantification, offering valuable insights and a potential framework for adapting similar approaches to IgE analysis (61, 62).

In a different approach, immunoaffinity capillary electrophoresis (IACE) coupled with Matrix-Assisted laser Desorption Ionization MALDI-MS was employed to diagnose cow's milk allergy by quantifying the Specific IgE in the serum of allergic patients (63). In MALDI-MS, the sample is co-crystallized with a matrix—a small organic compound that absorbs the laser energy, allowing for the gentle ionization of large molecules without fragmentation. Upon laser irradiation, the matrix absorbs energy and transfers it to the analyte, facilitating its ionization and subsequent detection by a Time Of Flight (TOF) mass analyzer (64). It is widely used in proteomics, biomarker discovery, and clinical diagnostics due to its high throughput, minimal sample preparation, and ability to analyze complex biological mixtures. Moreover, it is particularly advantageous for detecting intact proteins and post-translational modifications, making it a valuable tool for IgG quantification and characterization in serological studies (63).

Magnetic beads (MBs) coated with anti-IgE antibodies were used to isolate and quantify total IgE, providing a general allergy diagnosis. Subsequently, specific allergens, such as bovine serum albumin, Lactoferrin, and α-casein were identified as key allergens responsible for eliciting the allergic reaction (65). By chemically cross-linking the immunocomplex formed during the IgE quantification phase, these allergenic proteins were then detected using MALDI-MS, which directly identified the molecular masses and structures of the allergens. This method not only enabled precise identification of the allergens responsible for the allergic reaction but also allowed for the use of actual food extracts, which could lead to more tailored allergy diagnostics and epitope mapping. The detection was sensitive and required 2 µl of blood serum, making MALDI-MS a highly efficient tool in allergy diagnostics.

Similarly, Ultra-Performance Liquid Chromatography coupled with mass spectrometry (UPLC-MS) was recruited for the quantification of total IgE in human serum (66). The UPLC-MS uses columns packed with smaller particle sizes (typically ≤2 µm) and operates at higher pressures, leading to faster analysis, improved peak resolution, and greater sensitivity. When coupled with magnetic beads (MS), UPLC enables precise identification and quantification of small molecules, peptides, proteins, and metabolites (66).

**Figure 5:**
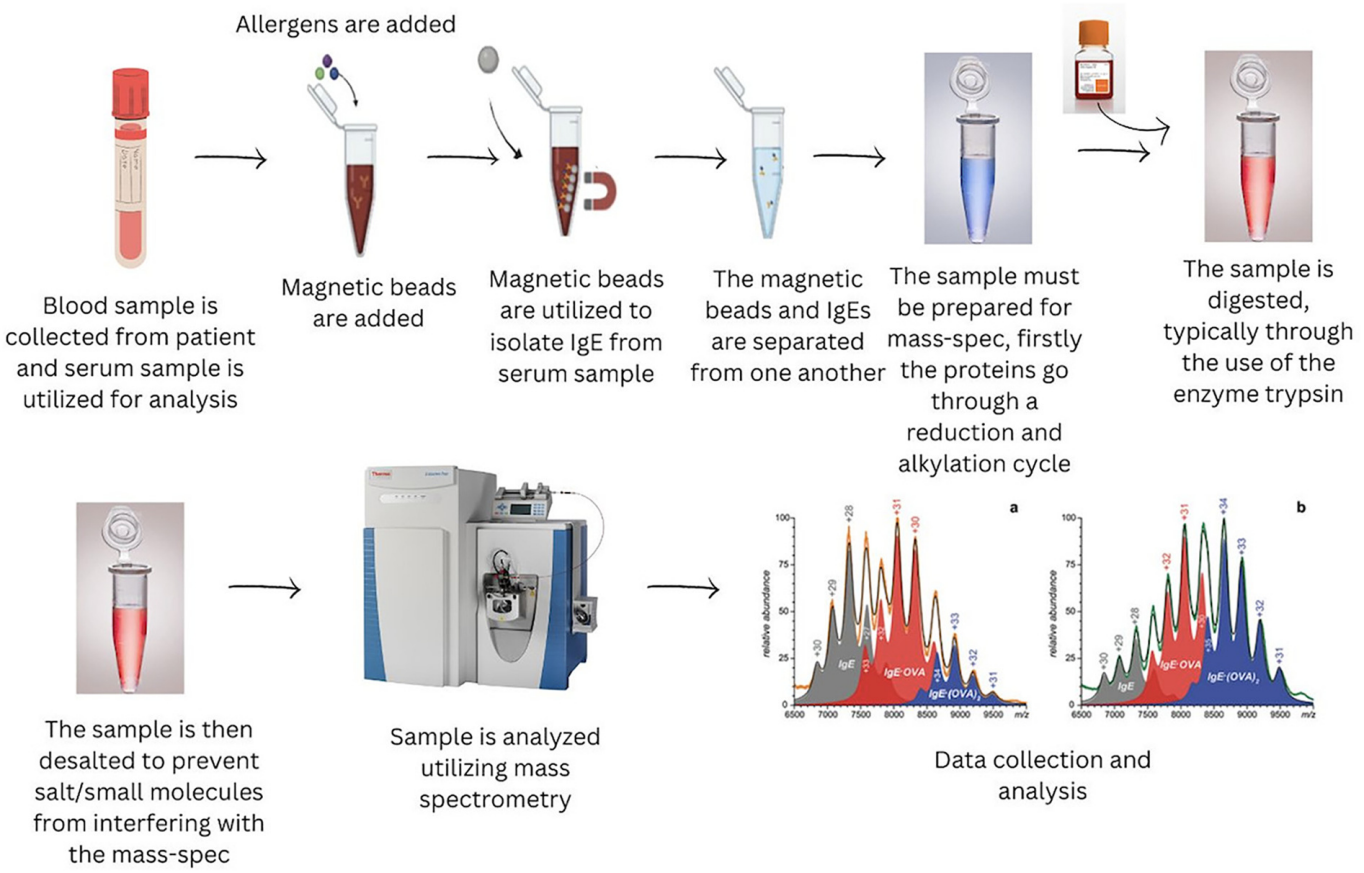
A workflow incorporating extraction of allergens with magnetic beads for UPLC –MS/MS analysis.

The MBs coupled with anti-IgE antibodies were used to extract and quantify IgE via a signal peptide after digestion. This method demonstrated high sensitivity, with limits of detection and quantification at 400 ng/ml and 800 ng/ml, respectively (66). By evaluating the binding capacity of the extracted IgE with different allergens, the study successfully identified allergens that induced allergic responses in patients. This MS-based method quantified total IgE and pinpointed the specific allergens involved, offering an advanced tool for allergy diagnosis with high precision. The method's effectiveness was shown by its application in analyzing serum samples from allergic and healthy individuals, further underscoring its clinical relevance in improving allergy detection and improving therapeutic decision-making. Figure 5 displays a general workflow for the clinical diagnosis of allergens, exemplifying advanced methodologies for sensitive and selective allergen detection utilizing MBs and UPLC-MS/MS technology.

### 3.2 Using MS to identify other early phosphoproteins in determining basophil activation

A critical component of the signaling pathway leading to basophil activation is spleen tyrosine kinase (Syk). Syk is essential for transmitting signals from the high-affinity IgE receptor (Fc*ε*RI) upon allergen binding, culminating in basophil degranulation and the release of inflammatory mediators (67, 68). Phosphorylation of Syk propagates downstream signaling events that result in calcium mobilization, degranulation, and the release of inflammatory mediators, ultimately driving the basophil's role in allergic responses. However, studies have discovered that a subset of individuals, referred to as “non-releasers,” possess basophils that fail to release histamine upon IgE receptor cross-linking due to the absence or significant reduction of Syk protein expression. This deficiency impairs the Fc*ε*RI signaling pathway, leading to false-negative results in BAT and challenging the reliability of standard basophil-based allergy diagnostics for these individuals (69, 70). To overcome these challenges, alternative methods have been explored to assess basophil activation in Syk-deficient individuals. One approach involves using non-IgE-dependent stimulants, such as *N*-formyl-methionyl-leucyl-phenylalanine (fMLP) or interleukin-3 (IL-3), which can activate basophils through alternative pathways (71). Additionally, measuring intracellular signaling markers like p38 MAPK and ERK1/2, or assessing the release of mediators such as histamine or leukotriene C4 (LTC4) by MS, may provide alternative indicators of basophil responsiveness (72, 73). Furthermore, ex vivo whole blood stimulation assays using cytokines like IL-3 or IL-33 have been proposed to characterize basophil activation when standard BAT markers fail. These alternative methods aim to provide a more comprehensive assessment of allergic responses, especially in individuals with atypical basophil activation profiles (74).

### 3.3 Quantifying signaling thresholds and post-translational modifications in basophil activation research using MS

One of the most promising applications of MS in basophil activation research is its capacity to quantify signaling thresholds and post-translational modifications (PTMs) of key regulatory proteins. A critical target in this context is CD45, a transmembrane protein tyrosine phosphatase that modulates Src family kinase (SFK) activity and plays an essential role in determining basophil activation potential. Dysregulated CD45 function can disrupt phosphorylation cascades downstream of Fc*ε*RI engagement, potentially leading to hypo-responsiveness in certain individuals. By utilizing MS-based phosphoproteomics, it is possible to detect differential phosphorylation patterns of CD45, providing mechanistic insights into its role in either facilitating or inhibiting early basophil activation events (75, 76).

Immunoreceptor Tyrosine-Based Activation Motifs (ITAMs) are conserved sequences found in the cytoplasmic tails of various immune receptors. They play a crucial role in signal transduction by recruiting and activating tyrosine kinases upon receptor engagement. MS offers a highly precise approach for characterizing ITAMs within Fc*ε*RI-associated signaling complexes, which play a crucial role in initiating allergic immune responses. Also, ITAM phosphorylation acts as a key regulatory checkpoint, governing the recruitment and activation of Syk kinase, a central mediator of intracellular signaling cascades downstream of Fc*ε*RI engagement. Disruptions in the phosphorylation dynamics of these motifs can significantly alter downstream signaling, contributing to immune dysregulation and hypo-responsiveness in allergic conditions (35, 77, 78). By leveraging targeted MS techniques such as selected reaction monitoring (SRM) and parallel reaction monitoring (PRM), researchers can achieve highly sensitive and quantitative measurements of ITAM phosphorylation states. This enables the detection of subtle alterations in Fc*ε*RI-mediated signal transduction, particularly in cases where basophils exhibit reduced responsiveness. Such quantitative insights are invaluable for identifying regulatory disruptions that may underlie immune tolerance, aberrant signaling in allergic diseases, or therapeutic resistance in conditions modulated by Fc*ε*RI activation.

### 3.4 Expanding the scope: early phosphoproteins in basophil activation

Beyond ITAM phosphorylation, MS can be employed to investigate a broader network of early phosphoproteins involved in basophil activation. LAT (Linker for Activation of T cells) and SLP-76 (SH2 domain-containing leukocyte protein of 76 kDa) serve as essential scaffolding proteins facilitating signal transduction from Fc*ε*RI to downstream effector pathways. Phosphorylation of these adaptor proteins plays a crucial role in orchestrating intracellular signaling events that determine basophil reactivity. Quantitative phosphoproteomics can be recruited to map phosphorylation sites on LAT and SLP-76, identifying alterations in their activation states that may contribute to immune dysregulation in basophilic non-responders (79–84).

Another key signaling molecule is PLCγ (Phospholipase C gamma), which is activated downstream of Fc*ε*RI signaling and is responsible for hydrolyzing phosphatidylinositol 4, 5-bisphosphate (PIP2) into inositol trisphosphate (IP3) and diacylglycerol (DAG) (85, 86). These second messengers are essential for calcium mobilization and protein kinase C (PKC) activation, both crucial for basophil degranulation. Using MS-based site-specific phosphorylation analysis, we can evaluate the activation state of PLCγ and identify potential dysfunctions that may lead to impaired histamine release and allergen-specific immune responses (80, 85, 86).

Moreover, Grb2-associated binding protein 2 (Gab2) functions as a docking protein that facilitates interactions with PI3 K (Phosphoinositide 3-kinase), influencing downstream Akt/mTOR signaling. Dysregulation of Gab2 phosphorylation may impact basophil survival and cytokine release, providing another avenue for MS-driven biomarker discovery. PRM-MS and phosphosite mapping could help elucidate changes in Gab2 phosphorylation that correlate with distinct basophil activation phenotypes (87–89).

### 3.5 Alternative activation pathways: IgE-independent basophil activation

Given that not all basophil activation is driven by Fc*ε*RI engagement, MS also plays a pivotal role in characterizing IgE-independent signaling pathways. Complement receptor C5aR (CD88) mediates basophil activation in response to C5a-C5aR interactions, serving as an alternative activation axis (90, 91). MS-based approaches allow for precise quantification of C5aR expression levels, the characterization of its PTMs (such as phosphorylation and glycosylation), and the assessment of downstream effector pathways. This is particularly relevant in individuals exhibiting non-responder phenotypes in the BAT test, where Fc*ε*RI-mediated activation is impaired, yet complement-driven basophil activation remains functional. By integrating quantitative proteomics, phosphoproteomics, and glycoproteomics, MS can provide a systems-level understanding of the molecular architecture underpinning basophil reactivity. These analyses are critical for refining diagnostic strategies and uncovering novel therapeutic targets in allergic diseases.

### 3.6 Kinase activity profiling in basophil signaling

MS-based approaches can be used for quantifying kinase activity within basophil signaling networks, enabling precise analysis of activation states and regulatory mechanisms. One particularly relevant kinase in this context is Lyn (Lck/Yes-related novel tyrosine kinase), a key member of the Src family kinases (SFKs). Notably, Lyn remains expressed at near-normal levels in non-releaser basophils, a subset of basophils that exhibit impaired degranulation despite activation stimuli (92).

Lyn plays a dual role in basophil activation, functioning both as a priming kinase that facilitates intracellular signaling and as a negative regulator that modulates Fc*ε*RI (high-affinity IgE receptor) and C5aR (complement component 5a receptor)-mediated pathways. Its ability to fine-tune these pathways is critical for maintaining immune balance (93, 94).

By utilizing phosphosite-specific enrichment (a targeted method for isolating phosphorylated peptides) in combination with tandem MS, LC-MS/MS), researchers can map Lyn activation kinetics with high resolution. This approach provides critical insights into dysregulated phosphorylation cascades, particularly in hypo-responsive basophils, where altered kinase activity may underlie impaired immune responses and allergic dysfunction.

Beyond Lyn, MS-based kinase assays can also assess activity levels of Syk, Btk (Bruton's tyrosine kinase), and PI3 K, which are key regulators of intracellular basophil signaling. Dysregulation of these kinases may contribute to impaired degranulation, cytokine release, and cellular priming, offering potential biomarkers for distinguishing between intrinsic activation defects and pathway-specific hypo-responsiveness (95).

### 3.7 MS in cytokine receptor signaling and alternative immune pathways

In addition to its role in receptor-mediated activation, MS can be used to quantify cytokine receptor signaling pathways involving interleukin-3 (IL-3), interleukin-4 (IL-4), and interleukin-5 (IL-5) (96, 97). These cytokines are crucial for basophil priming, survival, and effector function, and their receptors engage the Janus kinase (JAK)-STAT signaling cascade (98, 99). MS-based proteomic profiling facilitates: the quantification of receptor abundance to determine basophil responsiveness to cytokine stimulation, ligand-binding dynamics assessment to evaluate cytokine-receptor interactions, and downstream phosphorylation event analysis to identify molecular signatures of cytokine responsiveness. Such insights are particularly valuable for identifying alternative activation pathways in patients with atypical allergic phenotypes, where traditional IgE-centric diagnostics fail to capture the full spectrum of immune dysregulation. By expanding our understanding of basophil signaling networks through MS-based methodologies, clinicians and researchers can develop more comprehensive diagnostic tools and targeted therapeutic strategies to manage allergic and inflammatory conditions effectively.

## 4 Challenges and future directions of mass spectrometry in allergy diagnostics

Despite its immense potential in allergy diagnostics, the integration of MS into routine clinical workflows faces substantial technical, operational, and regulatory challenges that must be addressed to facilitate widespread clinical adoption.

One of the primary hurdles in applying MS to allergy diagnostics is the accurate quantification of proteins. Unlike traditional immunoassay-based techniques, such as ELISA, which measures total protein concentrations directly via antibody-antigen interactions, MS-based approaches typically rely on peptide-level quantification following enzymatic digestion (100). This introduces inherent variability due to factors such as differential peptide ionization efficiency, variations in post-translational modifications (PTMs), and proteolytic cleavage efficiency. These inconsistencies can lead to inaccurate quantification, which is particularly problematic in allergy diagnostics, where precise protein concentration measurements are critical for clinical decision-making (101).

To improve the reliability of MS-based protein quantification, absolute quantification techniques, such as isotope dilution MS (IDMS) and targeted multiple reaction monitoring MS (MRM-MS), have been developed. These methods incorporate stable isotope-labeled internal standards that correct for sample preparation and ionization variability (102). However, further refinement is needed to enhance sensitivity, reproducibility, and robustness, particularly for low-abundance allergenic proteins and IgE antibodies, which are often present in minute concentrations. Additionally, optimizing sample digestion protocols and developing improved enrichment strategies, such as immunoprecipitation-MS or affinity-based purification, could help mitigate issues related to peptide fragmentation variability and enhance detection sensitivity (103).

Mass spectrometers are inherently complex, requiring sophisticated hardware, precise calibration, and highly trained personnel for operation and data interpretation. Unlike immunoassays or flow cytometry-based techniques like the BAT, which are relatively straightforward to implement in clinical laboratories, MS-based workflows involve extensive sample preparation, advanced liquid chromatography separations, and rigorous bioinformatics processing. These factors significantly limit the accessibility of MS for routine allergy diagnostics, particularly in non-specialized clinical settings.

Automation and user-friendly software solutions are critical to overcoming these barriers. Advances in robotic sample preparation, microfluidics-based workflows, and artificial intelligence (AI)-driven data analysis platforms could simplify MS-based diagnostics and reduce reliance on highly trained personnel. AI-powered algorithms capable of automated spectral deconvolution, feature extraction, and real-time data interpretation could improve diagnostic accuracy and reproducibility. Furthermore, the development of ambient ionization techniques, such as paper spray ionization (PSI-MS) and matrix-assisted laser desorption/ionization (MALDI-MS), offers the potential for more streamlined workflows by reducing the need for extensive chromatographic separations and simplifying sample preparation.

For MS to be successfully incorporated into allergy diagnostics, standardization, and regulatory approval are critical (104, 105). Unlike conventional immunoassays, which rely on well-characterized antibodies and established clinical thresholds, MS-based approaches lack universally accepted reference materials, standardized protocols, and validated biomarkers for allergy-related proteins (106). This lack of standardization complicates inter-laboratory reproducibility and poses significant challenges for regulatory approval (107).

To address these challenges, global efforts should focus on developing certified reference materials for allergenic proteins and IgE antibodies, establishing harmonized sample preparation and analytical protocols, and implementing robust quality control (QC) measures across laboratories. Regulatory agencies, such as the FDA and EMA, will require comprehensive validation studies demonstrating the clinical reliability, sensitivity, and specificity of MS-based assays before approving their use in diagnostic settings. Collaborative initiatives between academic institutions, regulatory bodies, and industry partners will be essential in driving these standardization efforts forward.

The future of MS in allergy diagnostics lies in miniaturization, high-throughput adaptation, and integration with emerging technologies. One promising direction involves the development of portable and benchtop MS systems that can be deployed in clinical laboratories with minimal infrastructure requirements. Innovations in microfluidics and lab-on-a-chip technologies could further reduce sample volume requirements and improve assay throughput.

Ion mobility spectrometry (IMS), when coupled with MS, offers a refined analytical approach for distinguishing isobaric and isomeric ions-molecules with identical masses but differing in structure or conformation (108). This capability is particularly valuable in allergy diagnostics, where complex biological matrices often contain structurally related compounds that can confound accurate analyte identification (109). The IMS separates ions based on their size, shape, and charge in the gas phase prior to mass analysis, thereby enhancing molecular resolution and analytical specificity. This allows for more accurate detection and quantification of allergy-relevant biomarkers, such as specific immunoglobulins, lipid mediators, and signaling peptides implicated in hypersensitivity reactions. The incorporation of ion mobility into diagnostic workflows has the potential to significantly improve the accuracy, reliability, and clinical utility of MS-based assays in the evaluation of allergic diseases (109, 110).

## 5 Predicting severity of allergic reactions

BAT is a functional assay that measures the upregulation and activation markers, such as CD63 and CD203c, on circulating basophils following *in vitro* allergen stimulation. This test provides a direct assessment of immune cell activation, and its results correlate with the severity of allergic reactions observed during clinical food challenges. One of BAT's primary advantages is its ability to evaluate immediate hypersensitivity reactions, offering valuable insights into anaphylaxis risk. Additionally, it has shown a strong association between basophil activation levels and allergic reaction severity, making it a promising tool for identifying high-risk patients. BAT is also non-invasive and rapid, requiring only a small blood sample, with results available within hours. However, the test has some drawbacks, including the need for fresh blood samples, as basophils degrade quickly, necessitating same-day analysis. Furthermore, patient-specific variability influenced by factors such as medication use (e.g., antihistamines) and immune modulation can impact results. Additionally, standardization remains a challenge, as differences in laboratory protocols and reagents can affect reproducibility across institutions.

In contrast, MS-based allergy diagnosis offers a molecular-level analysis of allergic responses by quantifying allergenic proteins, analyzing immune complex formation (IgE and IgG-allergen binding), and profiling post-translational modifications (PTMs) that influence allergenicity. Furthermore, MS-based phosphoproteomic analysis can identify JAK-STAT and Fc*ε*RI signaling pathway activation, providing mechanistic insights into allergic severity. The advantages of MS include its ability to perform comprehensive molecular profiling, offering a detailed view of allergenic proteins, immune complexes, and intracellular signaling events linked to allergic responses. It is also highly objective and reproducible, as it eliminates variability due to cellular responsiveness, producing standardized quantitative data. Additionally, MS can identify phosphorylation patterns in effector cells, such as mast cells and basophils, which may serve as biomarkers for severe allergic reactions. However, MS has significant disadvantages, including high costs and the need for specialized instrumentation and expertise, limiting its accessibility in clinical settings. Furthermore, MS is not yet standardized for routine allergy diagnostics, as ongoing research is needed to validate its clinical utility. Another limitation is that MS does not directly measure real-time basophil degranulation or immune cell activation, as BAT does, but rather infers reaction severity through biomolecular signatures.

When comparing BAT and MS for predicting allergy severity, BAT is a functional immune assay that directly measures basophil degranulation and activation markers, while MS is a proteomic and phosphoproteomic tool that quantifies allergenic proteins, immune complexes, and signaling modifications. BAT has a fast turnaround time (hours), whereas MS-based approaches take days to weeks. BAT requires fresh blood with functional basophils, whereas MS can be performed on serum, plasma, or tissue samples. The challenges of BAT include variability due to medications, immune modulation, and sample stability, while MS is constrained by high costs, complex instrumentation, and lack of standardization. BAT is already used in some clinical settings for risk assessment, whereas MS remains primarily a research tool with the potential for precision allergy diagnostics in the future. Table 1 demonstrates a comparison of BAT vs. MS for Predicting Allergy Severity.

**Table 1:** A comparison of features between BAT and MS.

Feature	Basophil activation test (BAT)	Mass spectrometry (MS)
Type of analysis	Functional immune assay (measures basophil degranulation and activation markers)	Proteomic and phosphoproteomic analysis (quantifies allergenic proteins, immune complexes, and signaling modifications)
Prediction of reaction severity	Directly correlates with clinical severity in food challenges	Provides molecular-level biomarkers of severe allergic responses
Turnaround time	Hours	Days to weeks
Sample requirement	Fresh blood with functional basophils	Serum, plasma, or tissue samples
Challenges	Variability due to medications, immune modulation, and sample stability	High cost, complex instrumentation, and lack of clinical standardization
Clinical use	Used in allergy research and some clinical settings for risk assessment	Primarily a research tool with future potential in precision allergy diagnostics

In a nutshell, BAT is currently the more clinically applicable method, as it provides a real-time functional assessment of basophil activation and correlates well with clinical reaction severity. It is also faster, more affordable, and more accessible than MS, making it a valuable tool for allergy diagnosis and risk stratification. However, MS offers deeper molecular insights by profiling allergenic proteins, immune complexes, and phosphorylation signatures. While MS has the potential to identify novel biomarkers and enhance precision medicine in allergy diagnostics, its high cost and complexity currently limit routine clinical use. Moving forward, a combined approach that integrates BAT's functional immune assessment with MS's molecular precision may provide the most comprehensive predictive tool for assessing allergy severity. Standardizing MS-based biomarkers and making them more clinically accessible could revolutionize personalized allergy management in the future.

## 6 Sensitivity and selectivity across major allergy diagnostic tests

To date, MS is the least sensitive method for IgE detection, with a limit of detection (LOD) of 400 ng/ml (∼1,670 IU/ml), which is significantly higher than that of immunoassay-based techniques. In contrast, SPT and ELISA can detect IgE at levels as low as 0.35 IU/ml, making them far more sensitive for routine allergy diagnostics (66, 111). The most sensitive method, BAT, can detect IgE concentrations as low as 0.1 IU/ml, providing greater accuracy in identifying allergen-specific sensitization (39, 46). While MS offers high specificity and precise molecular characterization, its poor sensitivity limits its use for detecting low IgE concentrations in clinical allergy testing. Consequently, SPT and BAT remain the preferred methods for diagnosing allergies, as they are better suited for detecting and quantifying IgE at clinically relevant levels (46). Table 2 represents a Comparison of Minimum Detectable Concentration of IgE Tests.

**Table 2:** Comparison of minimum detectable concentration of IgE tests.

Test method	Minimum detectable concentration (MDC)	Strengths	Limitations
Mass spectrometry (UPLC-MS/MS)	400 ng/ml (∼1,670 IU/ml)	Highly specific, useful for allergen profiling	Less sensitive than immunoassays
Skin prick test (SPT)	0.35 IU/ml	Quick, cost-effective, widely used	Requires patient cooperation, affected by medications
Basophil activation test (BAT)	0.1 IU/ml	High specificity, detects functional IgE	Expensive, requires fresh blood
ELISA (enzyme-linked immunosorbent assay)	0.2–0.35 IU/ml	Widely available, semi-quantitative	Lower sensitivity than ImmunoCAP

## 7 Conclusion

Allergy diagnostics rely on a variety of methods, each with distinct advantages and limitations. The SPT and ELISA are widely used due to their cost-effectiveness and sensitivity, detecting IgE levels as low as 0.35 IU/ml. The BAT test offers higher specificity and provides functional and quantitative allergen-specific IgE data, making it a gold-standard technique for precise allergy profiling. However, these immunoassay-based approaches, while highly sensitive, may suffer from cross-reactivity and variability in results across different platforms.

Mass spectrometry has proven its value in allergy diagnostics through various applications, particularly in identifying and quantifying allergenic proteins in food and immunoglobulins in biological samples. Several studies highlighted the potential of MS to significantly enhance allergy diagnostics. However, further research is necessary to increase the practicality of MS and optimize its techniques for broader clinical applications. Although early studies have demonstrated its ability to quantify allergen-specific IgE and IgG, translating these methodologies into routine diagnostic tools for diverse allergic conditions remains a challenge. Future efforts are likely to focus on improving the sensitivity and throughput of MS technologies for detecting low-abundance targets including IgEs and addressing issues related to sample preparation and matrix effects. Despite these hurdles, integrating MS into clinical practice holds great promise. By integrating MS into existing diagnostic workflows alongside SPT, BAT, and ImmunoCAP, clinicians could achieve a more comprehensive, accurate, and personalized assessment of allergic responses, ultimately improving patient management and risk assessment.
